# American Academy of Dental Science

**Published:** 1899-01

**Authors:** 

**Affiliations:** American Academy of Dental Science


### AMERICAN ACADEMY OF DENTAL SCIENCE.

Dr. George S. Allan read a paper before the American Academy of Dental Science, May 4, 1898, entitled "Independent Journalism from a Dental Stand-Point."

(For Dr. Allan's paper, see Vol. XIX., page 761.)

### DISCUSSION.

*Chairman Pond.*—In the discussion of any question involving our advancement as a profession we have often received words of encouragement from members of a profession which always stands for the right. We have with us to-night a gentleman whose studies of the Puritans in England and New England will give added weight to his opinions with us, of New England birth. I refer to the Rev. E. H. Byington, D.D., of Newton.

*Rev. E. H. Byington.*—It will not be possible for me to add anything of importance to the very interesting discussion which we have listened to. I find that I did not understand at all what was meant by "independent journalism." I supposed that "independent journalism" meant something like the independent journalism which we have in the city of Boston, in a paper like the *Boston Herald*, which is more or less independent of all political parties, but not by any means independent of its subscriptions. The editorials and whatever is of interest to the reader is supposed to be unbiassed and independent in character. I find, after hearing the paper, that you are dealing with a subject which is very much more scientific and genuine.

I could almost have thought, while the paper was being read, that I was in a meeting of ministers, so high was the ethical tone of the paper, so constant and careful was the reference all along to the highest moral principles, and so desirous was the speaker to set forth the truth in regard to the matter. The fact is, gentlemen, in the profession which I represent we have a good many questions that are constantly coming up very much like this question that is before you. There are depots and bases of supplies which are closely connected with the theological profession, and there are sometimes presented in religious journals articles which, when the truth comes to be known, are found to have been shaded by mercenary motives; and it is not possible, even among ministers, to avoid those tendencies to which the selfish instinct within us constantly exposes us.

I wish you great success in the effort that you are making to bring the very highest principles into all these matters relating to your most useful profession, and I hope that in my own profession we shall be able, if not altogether to eliminate this tendency to pecuniary motives, at least to hold the evil influence in check.

*Chairman Pond.*—I am sure all of the older members of this society remember Dr. H. C. Meriam, who was formerly one of our prominent members, and you also know the stand he has always taken in matters of this kind. We have Dr. Meriam with us tonight, and would be very glad to hear from him.

*Dr. Meriam.*—It is rather a hard thing for me to say anything new on this subject. The president kindly alludes to a strong stand that I took in some previous years. I can assure him that in the semi-retirement that I have enjoyed for the past few years it has often seemed to me that I was entitled to a good deal of credit for not saying "I told you so." But the time has gone on, and I think that every one who has really interested himself has noted with great satisfaction the progress which has taken place in this direction. Mr. Whittier once said that he could conceive of nothing better for any young man than early in life to connect himself with some unpopular or neglected cause and press it to the front, and stick to it until it received the attention that the world would not otherwise give. There has been a gradual recognition of the fact that if dentistry is to be a specialty of medicine, it must have the same means of independent expression which the other specialties have. I venture to say that there is no specialty of medicine where the members do so much work, where they pay so much for the materials which they use, or where their work is done with such a strain on physical health and vigor.

You may remember that in an earlier paper—I think the very first paper that I wrote on this subject—I said we had no reason to complain of the shops. They were the delight of student days, and even now we can exclaim, as did Goldsmith of his muse,—

> "Thou source of all my bliss and woe,
> That found me poor at first and keeps me so."

And right here we touch again the side of the intellectual enfeeblement of our profession. The man who to-day can stand in any one of our shops and say that the dental materials are in advance of those of any other profession is an ignorant man. Let him but step into the shops where are exhibited scientific, electrical, surgical, or chemical apparatus, and he will find things that are infinitely more wonderful than are offered in our shops. I am sure that statement will be fully corroborated by Drs. Allan and Andrews.

Our duty is to keep an open door so that all progress relating to anything we need to do or use is not impeded. There is a tendency to teach in the dental schools the use of only such things as can be bought ready made, so that the students have a limited idea of the resources open to them. To buy ready made things and then trim the patient until they fit does not broaden men or develop their reasoning powers.

*Dr. Potter.*—It may be known to some of you that a few of us have been off on a short visit to New York. It has not been simply for pleasure. We had a very important meeting there, which was arranged by the Institute of Stomatology. We devoted a whole afternoon and evening to the discussion of professional morals, professional atmosphere, and all those things which Dr. Allan has summed up in his paper. I consider it is one of the most important dental meetings I have ever attended. At the close of the meeting there were a set of resolutions proposed and adopted as expressing the sense of the meeting on these important matters, and later the chairman, Dr. Bogue, asked me to bring this set of resolutions to the Academy and read them, to show what the Institute of Stomatology had been thinking about and doing, and with the hope that these resolutions might find a ready response in this society. I think I cannot do better than to read the resolutions at this time.

Dr. Potter then read a series of resolutions adopted by The New York Institute of Stomatology, May 3, 1898, and printed in the INTERNATIONAL DENTAL JOURNAL, July, 1898.

*Dr. Andrews.*—I make the motion that those resolutions be adopted as the sense of this Academy.

Unanimously voted.

*Chairman Pond.*—There is one more gentleman whom we are always glad to hear from, and who always takes an advanced stand on questions of this kind. I refer to Dr. Charles A. Brackett.

*Dr. Brackett.*—I feel like expressing a somewhat different view regarding the dentist—his moral quality, his position in the community, the way he is looked upon by his fellows—from that which the paper presents. Without any disposition to detract from any other profession, I do not think the dentists of this city, the dentists of this commonwealth, of New England or New York, are men who occupy a lower moral plane, with less thoroughly ingrained genuine morality, with a lower standard of ethics, personal or professional, than are the holders of the M.D., or other professional degree. Now, in the city in which I am a resident we have a worthy group of medical practitioners who deserve and command the respect and confidence of the community. We have a good number of practitioners of dentistry whose standing in the community is as honorable as that of the practitioners of medicine. I do not think any patient of any dentist in the city of Newport going to another dental practitioner would receive from any one of the twelve or fifteen located there any advice, hint, or insinuation that would not be honorable. I do not think there would be any attempt to hold out any inducement to continue in his charge. I

think there would be more readiness to advise the patient to go back to the dental adviser to whom he or she had been accustomed to go. I do not think our code of ethics is wrong. I do not think the nature of the practice of a dentist, having as much as any professional man can have the need of blind, unquestioning confidence on the part of those whom he serves, will admit of the moral standard being in any sense lower than that of any other body of professional men.

I expected that the essayist would have something to say in regard to our personal duty in connection with independent journalism. He has not come here with any word of upbraiding, such as "You have not stayed up our hands," or "You have not done for us as we expected," and yet I hoped he would point out some of the things that we ought to do to favor progress in the right direction. Too many of us are not as helpful as we should be in sustaining our journal. It would be well for us to ask ourselves, "What can I do to assist in advancing this cause?" One of the essential helps for every enterprise is money. A good share of the income of most periodicals comes through the advertising. As has been well suggested by our friend from Salem, there are many sources other than the special dealers from which we may advantageously obtain an extended variety of supplies. We should use our influence to have those who can furnish materials, tools, and instruments useful for us make themselves and their wares known through the advertising pages of our journal. The advantage would be mutual.

The INTERNATIONAL DENTAL JOURNAL has always had high merit in its reports of society meetings and in its extended original articles. It has been said that it might do better in having, in addition, more short, simple items not requiring much energy to write or to read, but which would be practical suggestions. It is in the power of every dentist to make, within the compass of a few lines, a half-page, more or less, reports of cases, suggestions about doing certain things and overcoming difficulties, that would be useful to others. If we will, each one of us, two or three times a year, contribute ten lines to the JOURNAL, the aggregate will be a large mass of material that will increase its attractiveness and be a great benefit to all concerned.

*Dr. Briggs.*—I was very much interested in Dr. Allan's paper, and heartily concur with him in all that he has said; and it seems to me that anything that we could do to prevent the domination of the trade journal is of the greatest advantage. The question of the ways and means of doing it is a complicated one, and I do not intend to go into it; but there is one point in particular that I want to bring out, and that is in regard to the education of the dentist. If he starts with an education as an all-round man, dealing with a specialty of medicine, the thing will work itself out in time.

Another thing I wish to refer to is the domination, the control, and the dependency of the dentist on the dental depot, and there again the education of the dentist comes in. It is simply the habit of following in the ruts of custom or tradition that has allowed men of the dental profession to be led about, dominated, if you please, by the dental depot. I have had that same thing said to me that Dr. Allan remarked as having been said to him,—"What would you dentists be if it were not for what we have manufactured for you?" It is a fair sample of what we as dentists may have said to us. It does not take much intelligence to see that we have made the manufacturing companies, and not they us. At the same time, we are to blame for the way that we allow them to dictate to us, and, as it were, adopt us, tell us what we want, what we should do, and what we shall have. I think, perhaps, we lack a little dignity in our dealings with them.

*Dr. Fillebrown.*—Dr. Allan referred to the means of remedying this condition. I think he made a good point in quoting the Century Dictionary. I think that definition is in accordance with the general public estimation of the dentist, and it is also true that the medical profession judges the dental profession in the same way. There are notable exceptions in both cases, of course. The paper also alluded to the fact that if the medical profession could know more of dentistry, that there would be the commencement of the remedy. It seems to me that this is not only the beginning of the remedy, but it is the one essential feature of it. I am glad I have an opportunity to talk to medical men about this matter to-night. I wish to call attention to and emphasize the ignorance of the medical profession in regard to the diseases of the human system which are dependent upon the teeth and their conditions. There is a most lamentable ignorance in that direction. This is not a reckless statement. My relations to the surgical clinic in the Harvard Dental School has brought this fact very strongly to my attention. I have known cases in the hands of some of our best surgeons in New England which have failed to be cured, but which have been readily cured at our clinic. One of them was a case of a patient with disease of the antrum that had rendered him utterly incapable of doing business. His antrum had been opened two or three times and treatment prescribed, but no relief was obtained until he came to our clinic. Eight badly abscessed roots were found, one or more of the abscesses discharging into the antrum. The removal of these roots and the treatment of the antrum enabled the patient to resume his work in a short time. Now, if that surgeon had known one-quarter as much about the teeth as he did about the other parts of the body, he would not have made that mistake.

An eminent surgeon took a man suffering from an abscess caused by the root of an inferior molar,—and only half a root at that,—and operated on him twice for necrosis, leaving big scars on the jaw. That patient finally came to what medical men regard as a second-rate institution,—a dental infirmary,—and received complete relief. So long as such ignorance prevails we cannot expect medical men to understand the position of dentistry. I could multiply these two cases by twenty without any exaggeration whatever. If our medical schools will insist that physicians shall know as much about the relations of the teeth to the various parts of the human system and the influence of their diseased conditions as they do about the effect of other diseased organs on the body, we shall then have a physician who will respect dentistry, and who will be a great deal more of a man himself. So long as our medical schools ignore a part of the human system, and neglect to instruct their students as to what its relations are, just so long will this unsatisfactory state of affairs exist. This is what I have preached for twenty years. We should be educated as medical men are, and each student be given particular instruction in regard to the specialty he intends to follow. Now, if the Harvard Medical School will add to its curriculum instruction in regard to dentistry,—a thing which they ought in all common sense to do,—and shall insist on as good a percentage in that course as in other studies, the time will not be long coming when patients will suffer less and dentists will be respected according to their ability.

*Dr. Bradley.*—It was my intention not to say anything, but the remarks that have been made call to my mind the fact that in preparing for the alumni exercises next June the committee of management have arranged for a series of papers and discussions, and one member of that committee has this evening spoken to another member about writing a paper which will treat on ideas very similar to those suggested by Dr. Andrews this evening, such as professional etiquette, not only in relation to how we should conduct ourselves in meeting brother operators, but in relation to how we should meet our patients and talk with them in reference to their former dentists or professional advisers. And I venture to say that in the school with which the gentleman is connected there is much indirect teaching of professional ethics as to what the student or dentist should be, and the building up of a professional character. It is indirectly done, but I sincerely believe that a great deal of the best instruction is given indirectly. A young man sometimes takes up with suggestions that are made indirectly, when the direct instruction is neglected.

From the paper and the discussion which we have listened to this evening it appears that we are striving for the ideal; that no profession has attained to it, as we have learned from the remarks of members of the medical profession and of the clergy, but we are all striving for the ideal. We need a higher ideal of the laity, as well as for the professions. It will take a long time to inculcate among them those principles which are necessary to those wishing to become useful citizens and desiring that which is best for themselves. I am encouraged to think, from my observations in this direction, that the trend is towards a higher ideal,—to leave the lower standard and come up into a higher plane of living. I think the American Academy of Dental Science has been active in working towards this ideal. It is within a few months that resolutions, framed by a member of this society, were passed, condemning the advertisement in the professional journals of nostrums and secret preparations; and I am also persuaded to think that it was that action which has started this agitation in favor of independence in dental journalism. I therefore feel that there is encouragement for us to go forward in the work of striving for a higher ideal in our professional life.

My good friend, Dr. Brackett, asks what we can do to favor our progress in these matters. Just this: see that the colleges carry along with them in their instructions to their pupils this helpful higher influence, and when they graduate give them helpful, strong literature that will reflect all that is best and highest in our profession and keep the degrading influences in the background.

*Dr. Bradley.*—Mr. President, I wish to move that a hearty vote of thanks be extended to Dr. Allan for his admirable presentation of this subject.

Vote passed.

*Dr. Allan.*—I feel very much pleased, gentlemen, with the kind way in which you have taken my paper. I assure you most earnestly that I believe the main idea, the essential idea, is one that in the end will be adopted by the profession at large. I heartily regret that my good friend, Dr. Brackett, should have put a meaning on some few things in it which were so different from what I believe and from what I intended to say. I certainly would not have said anything to any one that would have carried with it the suggestion that I had lost my respect for my profession in the slightest degree. My knowledge of the profession and of the honorable stand which it has taken in all questions of professional ethics, the high-minded way in which the members deal with each other, would not allow my thinking for one moment in the way my friend has suggested. I therefore beg that the doctor will deal with me kindly in that important particular. I take it for granted that my language was a little blind to thus put me in the position of being misunderstood.

Having written my paper and read it over, I wanted to throw it into the waste-basket, and if I had had a little more time to write another I certainly would have done so. Perhaps the trouble was that I tried to present the whole subject to the profession in one short paper. I could touch only on a point here and there. What I said about trade influences, the depots of supplies, I think was wholly true,—I do not know that I would want to change anything in that regard. There is no question whatever but that the profession has been staggering under a load of trade influences that from the beginning of our course in this great country has been a burden, an incubus, and an obstacle in our progress in almost every direction. I am thankful to believe that the profession to-day recognizes that fact, and stands ready to throw the burden off just as soon as a possible way is open.

What I should have done, could I have rewritten that paper, would have been to have said just this: we are creatures of heredity and environment. The old German saying has given greatest importance to the first. It goes something like this: "In man's development, a child should be very careful in selecting his parents." He ought to be, but unfortunately it is not in his power. But the body of man is the resultant of two forces, heredity and environment. We cannot change our heredity and we do not want to. We are a component part of the great body of medicine,—that is, dentistry is simply a part of the healing art, which is a fact that is coming to be more generally understood, and in the end will be recognized as universally as the law of gravitation. What we are we must be, and in a short time those articles and discussions on such subjects as "being a trade" and "sufficient unto ourselves,"

and all that sort of trash, will be relegated to the waste-basket of useless thoughts and intellectual rubbish.

The idea I would press, were I to write on this subject again, would be that the environments of the dental student should be such that when he leaves his college where he has acquired his education in dentistry, he will take his position naturally, easily as a member of the medical profession. His specialty will be dentistry. The aim and object of which is the medical and surgical care of the teeth and their adjacent and relative parts, and in his studies he should grow up in the atmosphere of the profession of which he is as really a part as the oculist or the aurist. How is it now? I have taken particular pains to inquire and I find it far different. The student of dentistry does not grow up with the idea that he is to be governed by moral and ethical laws to the same extent as the student of medicine, nor to anything like it. He is taught to a great extent that dentistry is an art, a science, something related to medicine, but not medicine; he is to practise on different lines, in a different way, and the whole atmosphere in the dental colleges, so far as I know, is hurtful instead of helpful to the professional instincts of the student when he goes out into the world to practise his calling. Therefore, I say, there cannot be too careful attention, too earnest endeavor, given to this fact in our profession. The student must have his environment such that, when he leaves his institution of preliminary training he will take his proper position in the professional world.

When the student receives his diploma, that is not all. His education must continue. The practitioner cannot remain at one point, he must either go up or down, one way or the other, and the fountain-head of all his instruction, from the time he leaves college, that source from which he is to derive his intellectual food, is the dental literature that comes into his hands, and I regret to say that the major part of that is under the control of the dental depots, with the natural trade ways which they adopt in the conduct of their business. They have a perfect right to employ the means that are common to the business world; to put their advertisements where they will find customers; to buy up all the current literature obtainable, also patents by the half dozen or dozen relating to one subject, and to select whichever is most profitable for them to manufacture and pigeon-hole the rest, thus depriving the profession of hundreds of useful, important, valuable ideas, which if they had not been patented, but placed on the open market, would have been of the greatest help to us. I am not finding fault with them, nor do I for one instant claim that the general law effects us more than it does them. The special code is what I want to give emphasis to. The moral law is the same for every man, in whatever capacity he may be placed, but we have the express law which says that a man who works for humanity should not bottle up his thoughts or take a royalty on the product of his invention, but should give freely to his profession whatever thoughts, invention, or discoveries he may have made for the benefit of suffering humanity.

So, I say, keep this literature free, keep it in a position where it will not be influenced by pecuniary motives. Just in proportion as you respect yourself will your neighbors respect you.

William H. Potter,
*Editor American Academy of Dental Science.*

